# Supplementary material for: Effect of Oxyfluorination of PFA-Coated Metal Mesh with Superhydrophobic Properties on the Filtration Performance of SiO2 Microparticles
Source: Molecules. 2023 Mar 30;28(7):3110. doi: 10.3390/molecules28073110 (PMC10095667; doi:10.3390/molecules28073110)
Supplement: Supplementary file 1 [file molecules-28-03110-s001.zip › molecules-2275075-supplementary.pdf]

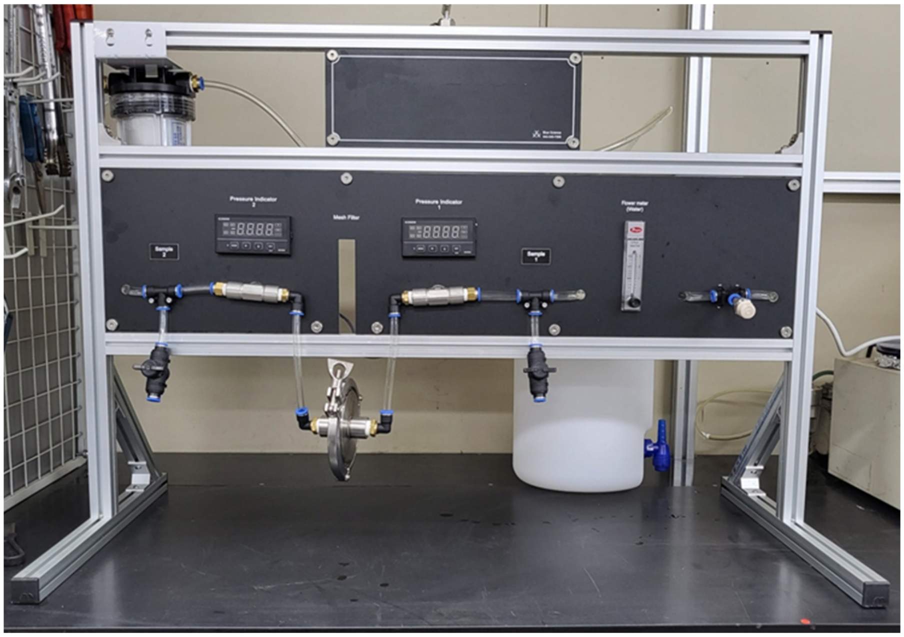

Figure S1. Experimental setup for the analysis of particle removal efficiency.

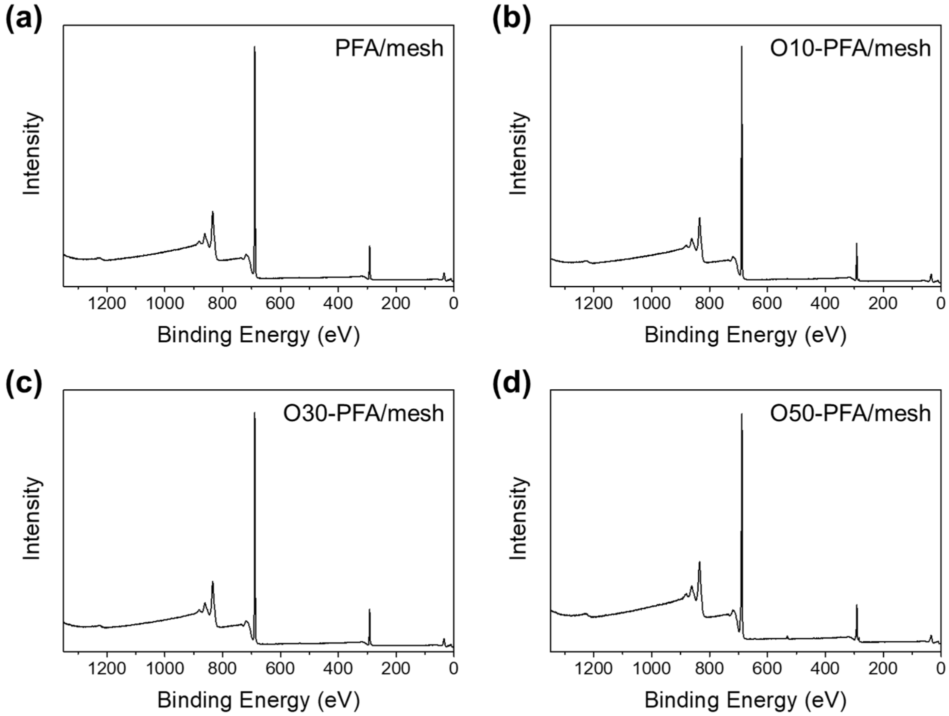

Figure S2. XPS survey of (**a**) PFA/mesh, (**b**) O10-PFA/mesh, (**c**) O30-PFA/mesh, and (**d**) O50-PFA/mesh.

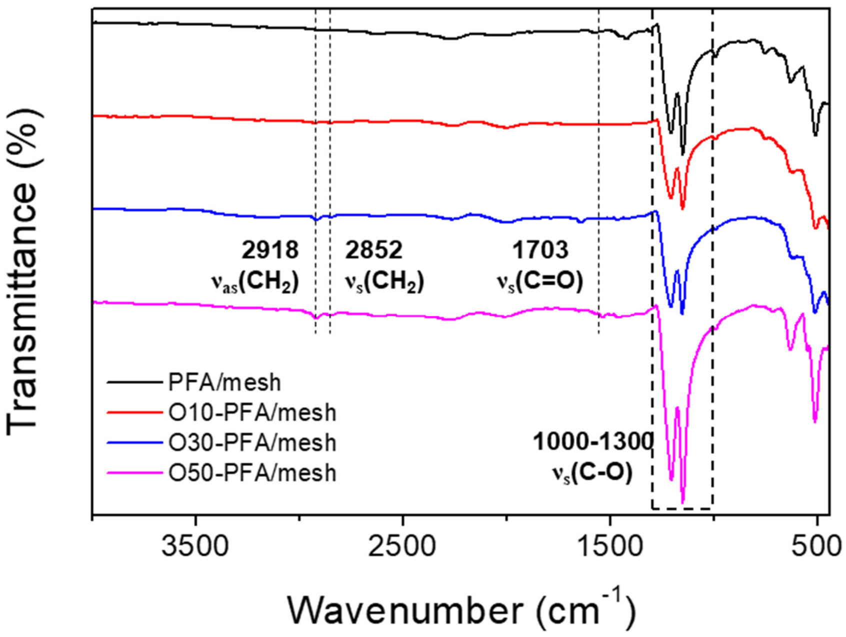

Figure S3. FT-IR spectra of PFA-coated mesh before and after surface modification by oxyfluorination.
